# Population-Based Prevalence of Intellectual Disability and Autism Spectrum Disorders in Western Australia: A Comparison With Previous Estimates

**DOI:** 10.1097/MD.0000000000003737

**Published:** 2016-05-27

**Authors:** Jenny Bourke, Nick de Klerk, Timothy Smith, Helen Leonard

**Affiliations:** From the Telethon Kids Institute, University of Western Australia (JB, NDK, HL), and Disability Services Commission, Perth, Western Australia, Australia (TS).

## Abstract

To investigate the prevalence of intellectual disability (ID) and/or autism spectrum disorders (ASDs) in Western Australia (WA).

A cohort of children born from 1983 to 2010 in WA with an ID and/or ASD were identified using the population-based IDEA (Intellectual Disability Exploring Answers) database, which ascertains cases through the Disability Services Commission (DSC) as well as education sources. Information on race, gender, mother's residence at birth and deaths was obtained through linkage to the Midwives Notification System and the Mortality Register. Diagnostic information on the cause of ID was obtained through review of medical records where available and children were classified as biomedical cause, ASD, or unknown cause.

An overall prevalence of ID of 17.0/1000 livebirths (95% CI: 16.7, 17.4) showed an increase from the 10-year previous prevalence of 14.3/1000. The prevalence for mild or moderate ID was 15.0 (95% CI: 14.6, 15.3), severe ID was 1.2 (95% CI: 1.1, 1.3), and unknown level of ID was 0.9 (95% CI: 0.8, 1.0)/1000 livebirths. The prevalence for Aboriginal children was 39.0/1000 compared with 15.7/1000 for non-Aboriginal children, giving a prevalence ratio of 2.5 (95% CI: 2.4, 2.6). Prevalence of all ASD was 5.1/1000 of which 3.8/1000 had ASD and ID.

The prevalence of ID has risen in WA over the last 10 years with most of this increase due to mild or moderate ID. Whilst the prevalence of ASD has also increased over this time this does not fully explain the observed increase. Aboriginal children are at a 2.5-fold risk of ID but are less likely to be accessing disability services.

## INTRODUCTION

Intellectual disability (ID) is a lifelong condition which impacts significantly on the individual and their family. The availability of reliable information on the number of individuals affected within a population is important to the planning and delivery of services. However estimates of the prevalence of ID in specific populations have been shown to vary widely from 2 to 21/1000 population.^1^ Factors affecting prevalence estimates include those related to the methodology of the study, such as the source of cases, the criteria used to identify ID and, the age-group included as well as those affecting the actual risk of ID, such as gender or sociodemographic status of the population.^1–4^ The prevalence of ID was found to be 18.3/1000 in a recent meta-analysis.^1^ The highest individual estimates were in studies of children and adolescents. Detection during adulthood tends to be less common due to fewer opportunities for diagnosis outside the schooling system and the likelihood of early death for those with more severe disability. Also, improved adaptive functioning during adolescence and early adulthood^5,6^ may also result in an individual with a mild ID being less likely to be diagnosed.

ID is characterized by impaired cognitive function and significant deficits in adaptive functioning, manifest before the age of 18 years.^7^ The clinical diagnosis of ID is generally supported using standardized measures of intelligence, which derive an intelligence quotient (IQ). For younger children unable to participate in comprehensive measures of intelligence, a broader measure of child development may be used. ID is defined as approximately 2 standard deviations or more below the population mean, which equals an IQ score of about 70 or below.^8^ This assessment is done in conjunction with adaptive functioning tests with a diagnosis of ID also requiring deficits of approximately 2 standard deviations in both measures, and onset in the developmental period. The prevalence of ID will vary depending on the case definition used to define eligibility and the cut-off scores applied.^2^ The classification of severity of ID has historically been defined in categories such as mild, moderate, severe and profound based on IQ scores with additional information of the intensity of support needs.^9^ However in more recent years the emphasis has moved from reliance on estimates of intelligence to a greater focus on the impact of ID on adaptive functioning. Whilst DSM-5 emphasizes the need to use both clinical assessment and standardized testing of intelligence when diagnosing ID, as was the case with DSM-IV, the severity of the ID in DSM-5 is based on adaptive functioning rather than estimates of intelligence alone.

For many people with ID, the cause may be due to an identified biomedical disorder, such as Down syndrome or Foetal Alcohol Syndrome, a disorder now being recognized and diagnosed more frequently in WA.^10^ However where no clear cause has been identified, as may be the case in over half of the cases of ID and up to three-quarters of those with mild ID,^11–13^ many possible factors may be contributing to the ID. These include social, behavioral, and educational risk factors.^7^ The increasing number of children being diagnosed with autism spectrum disorder (ASD) may also impact on the prevalence of ID, as they have been found to cooccur in approximately half of the cases,^14,15^ although a recent US study has shown the increase in autism rates to be greater for those without cooccurring ID.^14^ Whether the observed rise in autism is due to diagnostic transfer from ID has also been debated.^15^ The improved survival of preterm infants has been associated with the increased risk of insults to the central nervous system leading to a range of negative developmental outcomes which may include ID and autism.^16–18^

In Western Australia (WA) the prevalence of ID in the population cohort of children born from 1983 to 1992 was estimated at 14.3/1000 births.^11^ The aim of the present study was to investigate any change in the prevalence of ID in WA since the last study was conducted and to investigate trends to determine if the prevalence is increasing or decreasing.

## METHODS

Children born from 1983 to 2010 in WA with an ID and/or autism were identified using the IDEA (Intellectual Disability Exploring Answers) database.^19^ The main source of cases for the database is the Disability Services Commission (DSC), one function of which is to assist with the coordination of services for individuals with disability, including early intervention for ID and autism. Cases of ID were also identified through the Education Department of WA, with additional notifications sourced from Catholic Education and the Association of Independent Schools for those born 1983 to 1992, as described previously.^11^

Cases ascertained from the DSC were considered eligible for the study if they had a full scale IQ < 70 and met eligibility for ID^20^; they had a condition known to be consistent with ID (such as Down syndrome); or they had been documented as having ID after a review of medical files. Level of ID was defined as mild (IQ 55–69), moderate (IQ 40–54), or severe (<40). Cases identified only through Education sources were considered eligible if they had a level of ID defined as either mild or moderate, or severe. From 2006 onwards information provided on the severity of ID was replaced with a level of educational need (EN) coded as 1 (low need)–5 (high need). An analysis of the correlation between the previously assigned severity of ID and the level of educational need has shown that an EN score of 3 or 4 correlates highly with a mild or moderate ID (94.15%), and EN score of 5 correlates highly with a severe ID (86.42%) (Kendall's tau-b = 0.804). Therefore cases identified only through Education sources were considered eligible for the study if they had an EN level = 3 or 4 (coded mild or moderate) or 5 (coded severe). Level of ID for all cases was then coded into 1 of 3 categories as mild or moderate, severe or unknown.

The age at ascertainment was based on the year in which the child registered with DSC or for those known only to Education sources, the year in which they were first identified by the Department as requiring educational support. Information on date of birth, gender, race, and location at birth were provided through linkage to the Midwives Notification System and mortality information from the Deaths Register, using the WA Data Linkage System.^21^ For cases ascertained through DSC, diagnostic information was reviewed from medical records. Cases were classified as biomedical cause (e.g., chromosomal disorders, metabolic disorders, prenatal exposure to teratogens such as alcohol, postnatal injury), associated with ASD, other associated conditions (e.g., prematurity, birth complications) or unknown cause.^22^ In WA, diagnosis of ASD is based on the Diagnostic and Statistical Manual (DSM) classification^8^ and cases with a mild or moderate, severe or unknown level of ID were coded as having coexisting ID. For cases with a biomedical diagnosis cooccurring with autism, the biomedical cause took precedence for cases with a clear syndrome or diagnosis consistent with ID. No medical information was available for cases ascertained only through Education sources and these were assigned as unknown cause.

Prevalence of ID in any period was defined as the proportion of all children liveborn in WA and alive at the end of the period, who were diagnosed with ID by 2010. Total prevalence with follow-up to 2010 was estimated by limiting to birth years 1983 to 2005 to allow for a minimum of 5 years of follow-up, enabling ascertainment through education sources. Cumulative prevalence was calculated by progressively estimating the prevalence for births from 1983, with additional births from each subsequent year contributing to the prevalence, for birth years from 1983 to 2005. Prevalence was expressed per 1000 livebirths. Population data by gender, race, and location at birth and death information for birth years 1983 to 2005 were used for the denominators. Stata v13 was used for estimation of all prevalence ratios and 95% confidence intervals using the “csi” command. This procedure estimates standard errors using the method suggested by Katz et al.^23^

Ethical approval for this study was granted by the Western Australian Department of Health Human Research Ethics Committee (#2011/64). Approval to analyze the data by Aboriginal and Torres Strait Islander status was granted by the Western Australian Aboriginal Health Ethics Committee (#613).

## RESULTS

There were 721,645 children live born in WA between January 1, 1983 and December 31, 2010 of which 10,631 were identified with an ID by 2010. Of these 6953 (65.4%) were identified through the DSC and 3678 (34.6%) were unique to Education sources. There were 64.95% males and 35.05% females. Of the 10,631 there were 330 who had died by 2010. In addition to those with an ID, there were 675 diagnosed with an ASD and no ID.

**FIGURE 1:**
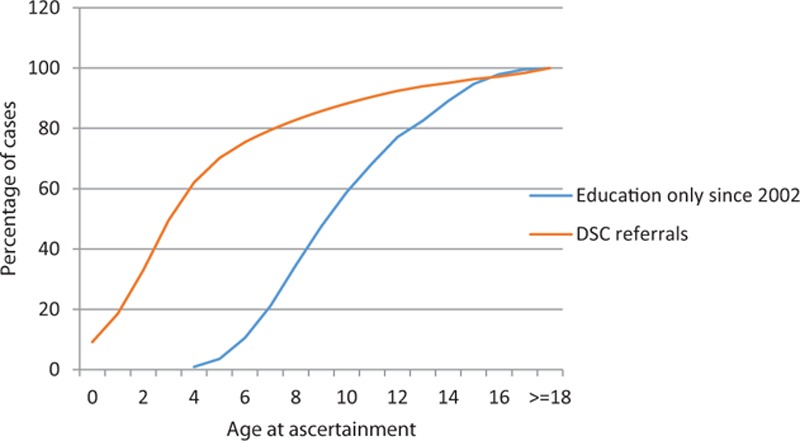
Age at ascertainment by source.

The age at case ascertainment either through DSC or only through Education sources is shown in Figure 1. As the first cohort of children ascertained through education sources was provided in 1999 for births 1983 to 1992, without a true age at ascertainment, only cases notified at the subsequent updates in 2002 and later were plotted. By 6 years of age, 75% of those identified by DSC had registered, whereas 75% of those identified only by education were not known until 12 years of age.

## PREVALENCE

**FIGURE 2:**
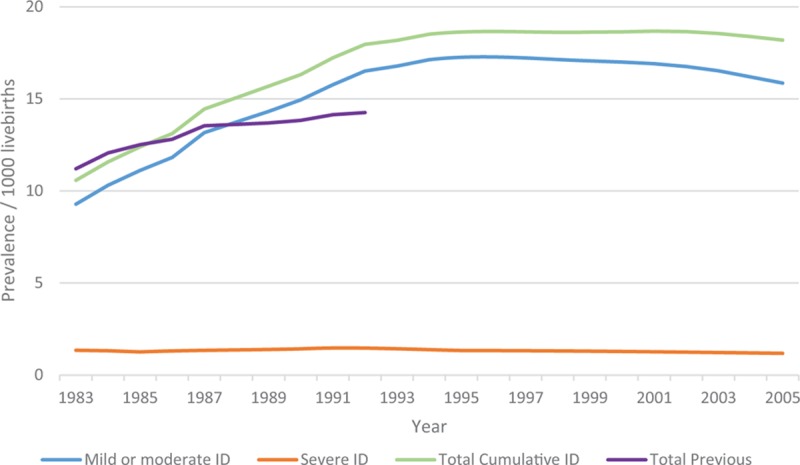
Cumulative prevalence for births 1983 to 2005 with follow-up to 2010 by severity of intellectual disability (ID). Note: Unknown level of ID is included with mild or moderate ID. Total previous prevalence is for births 1983 to 1992 with follow-up to 1999.

For births 1983 to 2005, with follow-up to 2010 (n = 9625), there was a total prevalence of ID of 17.0/1000 livebirths (95% CI: 16.7, 17.4). The prevalence for mild or moderate ID was 15.0 (95% CI: 14.6, 15.3), severe ID was 1.2 (95% CI: 1.1, 1.3), and unknown ID was 0.9 (95% CI: 0.8, 1.0)/1000 livebirths (Table 1). Cumulative prevalence over the period 1983 to 2005 is shown in Figure 2 by level of ID. These compare with previous estimates for WA births 1983 to 1992 with follow-up to 1999 of a total prevalence of 14.3/1000 births, 10.6/1000 for mild or moderate ID, 1.4/1000 for severe ID, and 2.3/1000 for unknown ID^11^ and represent an overall increase in prevalence of ID of 19% from 1999 to 2010.

**TABLE 1:**
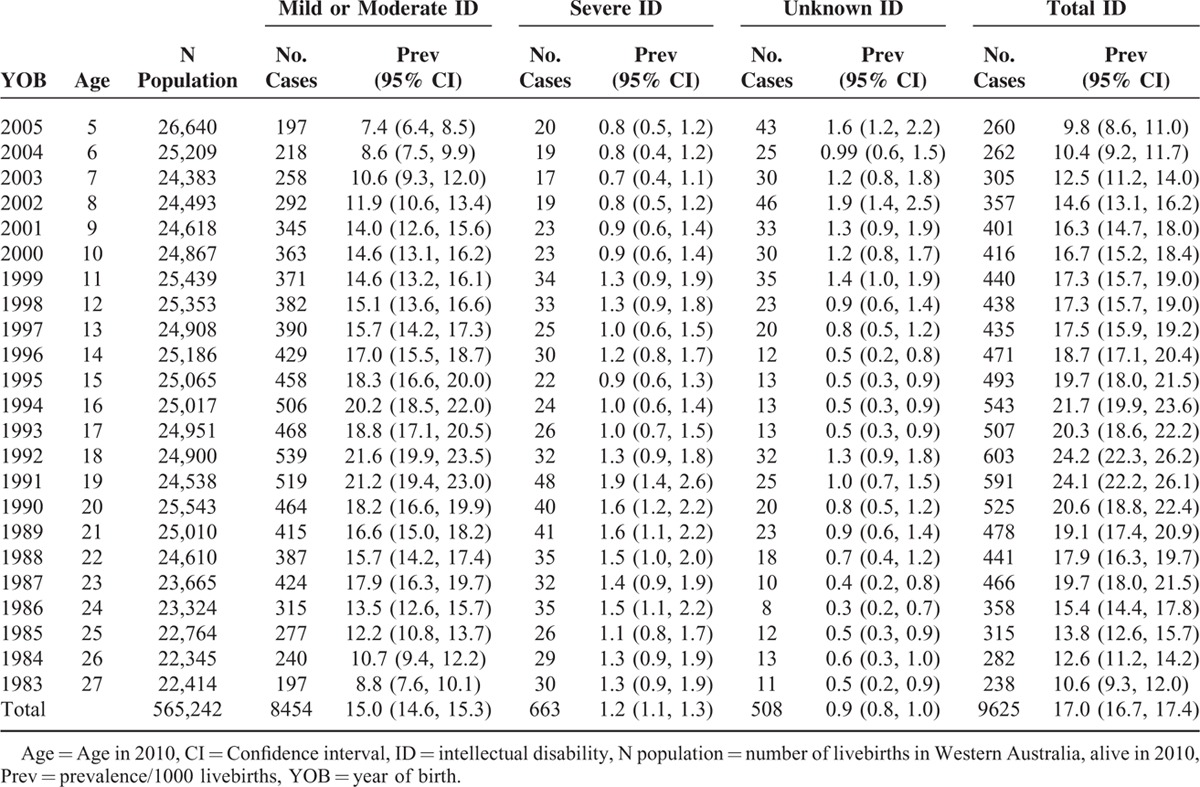
Prevalence of Intellectual Disability for Births 1983–2005, With Follow-Up to 2010

The prevalence was 21.7/1000 for males and 12.2/1000 for females, prevalence ratio 1.78 (95% CI: 1.71, 1.86), which was similar for both sources of ascertainment (Table 2). The prevalence for Aboriginal children was 39.0/1000 compared with 15.7/1000 for non-Aboriginal children, prevalence ratio 2.49 (95% CI: 2.35, 2.64). However for ascertainment through DSC the prevalence ratio was 1.24 (95% CI: 1.12, 1.37) compared with 4.95 (95% CI: 4.59, 5.34) for ascertainment through education sources (Table 2). The increased Aboriginal prevalence was highest for those with mild or moderate ID and to a lesser extent for those with severe ID (prevalence ratio = 2.65 and 1.67, respectively) (Table 3). Compared to metropolitan residence (prevalence 15.6/1000), the prevalence was increased in all regional or remote areas with the highest in very remote areas being 18.9/1000 (Table 2). However this increase in regional and remote areas was not evident for those ascertained by DSC, with a prevalence ratio from 0.93 in outer regional to 0.80 in very remote regions. The increased prevalence in regional and remote areas was also only evident for those with mild or moderate ID (Table 3).

**TABLE 2:**
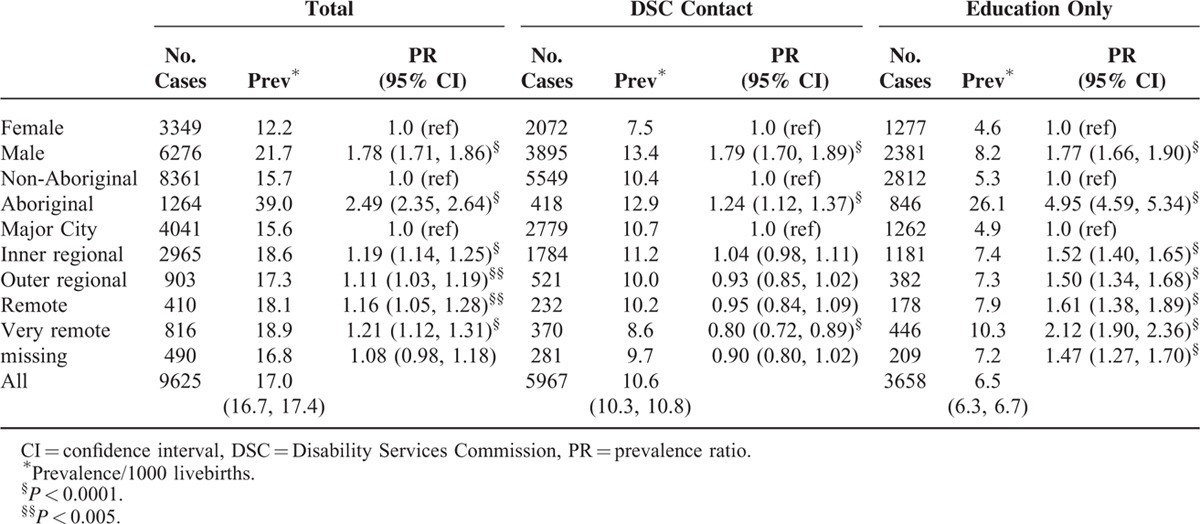
Prevalence of Intellectual Disability in Western Australia by Gender, Maternal Race, and Place of Residence at Birth, According to Source of Ascertainment, for Births 1983–2005 Ascertained to 2010

**TABLE 3:**
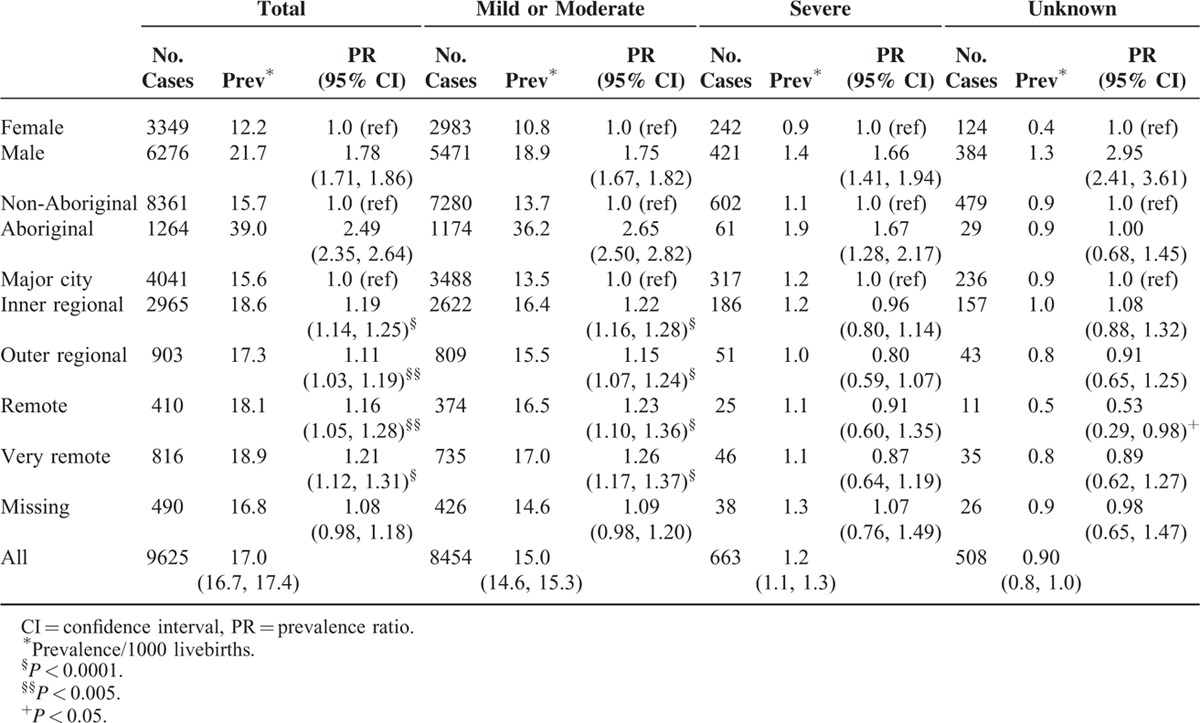
Prevalence of Intellectual Disability in Western Australia by Gender, Maternal Race, and Place of Residence at Birth, According to Source of Ascertainment, for Births 1983–2005 Ascertained to 2010

There were 2307 children born 1983 to 2010 and alive in 2010 who were diagnosed with an ASD. Of the 2307, 675 (29.3%) definitely did not have an ID. The total ASD prevalence was 4.1/1000 births, with 2.6/1000 with an ID and 1.2/1000 with no ID. Total ASD increased from 0.7/1000 for 1983 births to a maximum of 6.7/1000 for 1997 births, before declining to 3.3/1000 for 2005 births. The establishment in WA in 1991 of a multidisciplinary Central Diagnostic Panel which was set up to conduct the diagnostic assessment of autism, coincided with a marked increase in the annual incidence of autism.^24^ A further marked increase was observed when early intervention funding for autism was set up in 1997.^15^ Limiting to birth years 1993 to 2005, when the diagnostic processes for ASD became more regulated, shows a prevalence of all ASD of 5.1/1000, of which 3.8/1000 had ASD and ID. The prevalence of ASD with ID was previously estimated in 1999 for births 1983 to 1992 as 0.8/1000 births.^25^

### Causes of Intellectual Disability

**FIGURE 3:**
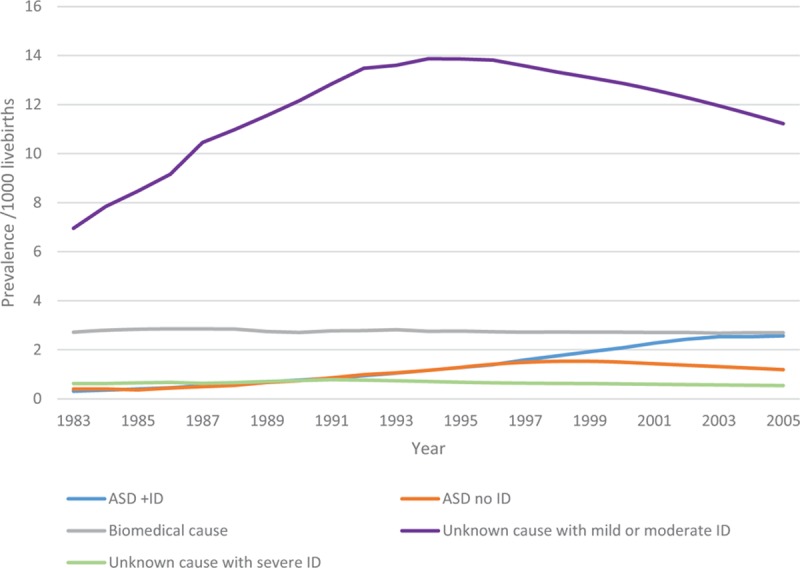
Cumulative Prevalence for births 1983 to 2005 followed up to 2010 by cause of intellectual disability.

For the 11,306 children born 1983 to 2010 and diagnosed with either ID or autism, 1955 (17.3%) had been assigned a biomedical diagnosis; 2307 (20.4%) had a diagnosis of ASD, of whom 1632 (14.4%) had comorbid ID; 2404 (21.3%) had an associated condition/risk factor such as prematurity, birth complications or epilepsy; and the remainder (41%) had no diagnosis. These included the 3678 children who were only ascertained through education sources where no medical information is provided. The most common biomedical diagnoses were Down syndrome (33.6% of all biomedical cases), other genetic (including chromosomal) abnormalities or birth defects (43.3%), infections (6%), maternal alcohol (5.1%), and postnatal injury (2.6%). Whilst ASD prevalence (both with and without ID) increased over the period, the prevalence of biomedical cause and of severe ID of unknown cause remained fairly constant (Figure 3). Mild or moderate ID of unknown cause has increased over the period and the decline in latter years is likely due to a delay in ascertaining children over 5 years of age.

## DISCUSSION

The prevalence of ID in WA has increased over the past 10 years compared with previous estimates. Most of the increase has been in those with mild or moderate ID. This increase is associated in a large part with an increased prevalence of ASDs for whom 70% had comorbid ID or an unknown level of ID. The prevalence of ID for Aboriginal children and young adults is 2 and a half times that of non-Aboriginal children and young adults.

The proportion of cases identified through the DSC has increased (65% compared with 45% previously) but those identified only by education sources are now even more likely to be Aboriginal. The two and half-fold higher prevalence of ID in Aboriginal children is similar to that previously reported in WA^11^; however, for those identified only through education sources this risk has increased from 3- to 5-fold. The widening of this disparity is concerning, as although children identified within the education system should be receiving support in this setting, they will not be receiving the early intervention services so crucial to a child's development.^26^ Our finding of the increased likelihood that Aboriginal children will have a mild or moderate ID may partly explain why these children may not be diagnosed until they are challenged within the school system. It may also be that parents are less likely to access early intervention services for their child due to less considered need for services, a lack of awareness of services or difficulties in navigating the disability support system. However, the association between lower socioeconomic status and increased rates of ID well documented here and elsewhere^3,4,25^ also helps explain the diminished access to these services by Aboriginal children. The increasing awareness and diagnosis of Fetal Alcohol Syndrome (FAS) in WA^10^ suggests that a proportion of the ID of unknown cause seen in our Aboriginal population and ascertained through education sources may be due to FAS.^27^

Changes in diagnostic criteria for ASDs from DSM-III to DSM-IV, increased awareness and acceptance of this diagnosis as well as specific funding for early intervention have been shown to affect the prevalence of ASD^15,24^ and further effects are expected with the introduction of DSM-5.^28^ Our study reflects the observed increase in ASDs seen in other countries^29–33^ as well as in WA.^15^ We estimated a prevalence of all ASD, both with and without ID, of 5.1/1000 in the latter period since 1993 when the introduction of a Central Diagnostic Panel brought a coordinated approach to the diagnostic process of ASDs. The increase we have observed in overall prevalence of ID may be partly attributable to an increase in ASD, which we calculated as 2.6/1000 for those with ASD and ID over the whole study period. The mechanisms for this possible contribution may be a real increase in ASD, where a proportion of cases will have ID; increased opportunity for diagnosis of ASD cases, in whom both ASD and ID would previously have been undiagnosed; or we may have incorrectly attributed the presence of ID to some individuals with ASD, whose intelligence was unable to be tested. However, it cannot fully explain the rise in ID which we have shown. It is possible that improved screening and diagnostic practices may have contributed to this increase. It is also possible that a proportion of the observed increase in mild or moderate ID may be attributable to un-diagnosed Fetal Alcohol Syndrome, as highlighted in a study of WA mothers hospitalized for alcohol-related disorders.^27^ This study conservatively estimated that about 4% of all nongenetic ID was related to heavy maternal alcohol use, with almost 16% attributable for Aboriginal children. The majority (over 90%) of the children had a mild or moderate ID.

With a greater number of infants now surviving very preterm birth^17^ and the increasing proportion of infants being born preterm between 34 and 38 weeks^34^ there is increasing evidence of poorer neurological outcomes^16,35,36^ as well as high hospitalization rates^34,37,38^ in these children. It is possible that the increase we have seen in the prevalence of mild to moderate ID may also be associated with increasing preterm birth rates and the associated neurodevelopmental consequences.

The main strength of this study is its use of a population-based database on children diagnosed with ID using psychological assessments based on a number of sources and its ability to observe trends over time. The linkage of population birth data provided information on race and location at birth in order to observe differences. A study investigating trends in the prevalence of specific developmental disabilities for 8-year olds in Atlanta, US over the period 1991 to 2010 using multiple education and health sources, found the prevalence of ID remained fairly stable over this time with an average of 13/1000.^33^ However, we found the majority (60%) of children ascertained only through education sources were not identified with ID until after 8 years of age which may explain our higher total prevalence of ID. The US study showed similar increased prevalence of ID in males and non-Hispanic Black children with the majority of children classified with mild ID (63.7%) compared with 87.8% with mild or moderate ID in our study.

A limitation of the study is that it may be missing a small number of cases which were ascertained through Catholic and Independent schools for births 1983 to 1992 but not for later birth years, although the number unique to this source was small (3.5%). The inability to obtain medical information on cases ascertained only through education sources is a limitation and therefore information on the associated causes of ID are limited to those accessing disability services. It is also likely that some children ascertained through the DSC in recent years may not have been fully investigated before registration such that information on the etiology of their ID was not available to the database. Only children with a known intelligence assessment of borderline or no ID were included in the ASD without ID group. Thus children with an autism diagnosis but an unknown level of ID were classified within the comorbid ASD and ID group. Therefore there may be some children within the ASD with ID group who will be subsequently found not to have an ID. Different categorizations of severity level of ID has been recognized as a methodological difficulty^8^; however, for this study it was necessary to combine the categories of mild and moderate ID into 1 group. This may make some comparisons with other studies difficult.

Prevalence studies on ID provide an important estimate of the depth of support that may be required within a community across many areas including disability services, health, education, and family support. The impact of caring for a child with ID or autism on parental well-being cannot be under-estimated^39–43^ and the provision of services for these families is an important component in enabling a better quality of life for their child.^44^

## CONCLUSION

Estimates of the prevalence of ID have risen over the last 10 years from 14.3/1000 to 17.0/1000 with most of this increase in the mild or moderate ID group. Whilst the prevalence of ASD has also increased over this time, estimated in the most recent years to be 5.1/1000 of which 3.8/1000 had comorbid ID, this does not fully explain the observed increase. Compared to non-Aboriginal children, Aboriginal children are at a 2.5-fold increased risk of ID but are much less likely to be accessing services through the DSC. It is possible the observed increase may be due to improved screening and diagnostic practices however a proportion of the observed increase in mild or moderate ID may be attributable to undiagnosed Fetal Alcohol Syndrome.
